# Abdominal Tuberculosis in Childhood

**Published:** 1905-06-17

**Authors:** 


### Hospital Clinics.

### ABDOMINAL TUBERCULOSIS IN CHILDHOOD.

In a paper read before the Medico-Chirurgical Society,[1] Dr. Branson gave the results of an investigation of 40 cases in which the condition was clinically a primary tuberculosis of the abdomen. He described the elementary lesions as consisting of three items, lesions of the intestines, lesions of the mesenteric lymphatic glands, and lesions of the peritoneum, any one of which may achieve an importance clinically paramount, though they generally occur together in varying degrees of prominence.

The lesion of the gut is ulceration, transverse to the long axis of the gut, and affecting the lower part of the ileum for choice. The serous face of such ulcers are generally the site of grey tubercles, and adhesion to neighbouring coils of bowel is therefore a common feature. As a rule the appropriate mesenteric glands show some degree of caseation.

The lesion of the mesenteric glands is caseation, and the adhesion of large masses of these glands to the anterior abdominal wall produces the pouting navel characteristic of the advanced disease. Some doubt exists whether or not mesenteric caseation postulates a previous lesion of the gut. It may be impossible to find one after death, but in general mesenteric caseation connotes intestinal ulceration.

The lesion of the peritoneum may be one of two main varieties—a general miliary tuberculosis or an adhesive peritonitis. The distinction is clinically sound, but all combinations may be seen in the dead-house. The plastic form occurred in the author's series about four times as often as the miliary, which is generally accompanied by ascites.

Pure tuberculous enteritis without gross lesions of the peritoneum is described as very rare, as is palpable enlargement of the mesenteric glands in the absence of peritoneal involvement, though it is suggested that more careful search would more often reveal the latter condition, which is not uncommonly found by accident in autopsies on children dying of an apparently primary tuberculous peritonitis. Of 31 autopsies on cases of this kind seven, or 22 per cent., showed no old lesion of tuberculosis elsewhere than in the intestines or mesenteric glands, which emphasises the importance of the condition.

The peritoneal cases, which form by far the clinical majority, are described as presenting two more or less defined periods, the first preceding and the second following the appearance of local symptoms and signs. The first period is one of vague ill-health and loss of flesh, generally associated with some irregularity of the bowels, though not in any fixed direction. The second period, or period of definite peritonitis, is heralded by distension of the belly. In the miliary form this distension depends to a variable extent upon serous effusion free in the abdominal cavity; in the plastic form it is due to interference by adhesions with the free peristalis of the gut, that is to a partial obstruction. It is probable, according to the author, that the real starting point of the second stage in plastic cases is parietal adhesion of the intestines, for parietal adhesion is much more likely to limit peristalsis than is inter-intestinal adhesion. That the period of distension is largely obstructive is shown by the frequency with which in plastic cases the distension of the belly is accompanied by abdominal pain and vomiting.

The earlier stages are frequently overlooked in hospital patients, and a child may be brought up for intestinal obstruction whose previous loss of health has not attracted the attention of its parents. A few cases are a-febrile throughout, but occasional rise is frequent even in mild cases, while high hectic fever is the rule in cases proving fatal.

Many ascitic cases pass later into the ordinary plastic form, after removal or absorption of the fluid. The ascitic stage is not of immediate gravity. In plastic cases retrogression of the disease is shown by gain in weight and the subsidence of grave symptoms like high fever or diarrhœa. The majority of plastic cases, however, gradually go down hill. In fatal cases death is commonly brought about by the exhaustion consequent upon diarrhœa and pain.

No case in the series was under the age of two, and 14 of the 40 cases were in their third year, which appears to be the year of choice for abdominal tuberculosis as a clinical condition. In six of eleven autopsies upon cases in the series the infection was limited to the abdomen, a result which is hard to reconcile with Koch's dictum that primary abdominal tuberculosis is a great rarity.

The diagnosis in the first stage can only be made if caseous glands can be palpated. In the second period the ascitic form has to be distinguished from cirrhosis of the liver. This is a rare disease in childhood, but when it occurs the liver is hard, whereas in the tuberculous disease it is commonly soft from fatty infiltration. The plastic form has to be differentiated from chronic gastro-enteritis of a simple kind, particularly that called mucous disease by Dr. Eustace Smith. Here again there is often no other point of distinction beyond the hard enlargement of the mesenteric glands in the tuberculous disease.

The outlook in this disease, based upon the cases of the series, appears exceedingly gloomy, among hospital patients at least. The figures were as follows:—

| | Per cent. |
|---|---|
| Total mortality of diagnosed abdominal tuberculosis ... | 71 |
| Mortality of plastic cases alone ... ... ... ... | 76 |
| Mortality of ascitic cases alone ... ... ... ... | 50 |

Eight survivors were examined. Three, who had been the subjects of the ascitic variety, appeared in good health after intervals ranging from ten months to three years. Of five survivors of the plastic form, seen at intervals ranging from 18 months to five years, two, though in fair bodily health, were profoundly tuberculous, one having many large glands on both sides of the neck, the other a large nodular mass in the belly. The remaining three seemed to be in excellent health, though two of them, girls, now aged 16, in spite of their present robust health, have never menstruated. If this should prove to be a constant result of the disease, another element of gravity is added to it.

[1] June 6, 1905.

